# Recombinant Myeloperoxidase as a New Class of Antimicrobial Agents

**DOI:** 10.1128/spectrum.00522-21

**Published:** 2022-01-12

**Authors:** Zehong Cao, Guangjie Cheng

**Affiliations:** a Division of Pulmonary, Allergy and Critical Care Medicine, Department of Medicine, University of Alabama School of Medicine, Birmingham, Alabama, USA; Georgia Institute of Technology

**Keywords:** HEK293 cells, myeloperoxidase, therapy, animal models, antimicrobial activity, recombinant-protein production

## Abstract

Heme-containing peroxidases are widely distributed in the animal and plant kingdoms and play an important role in host defense by generating potent oxidants. Myeloperoxidase (MPO), the prototype of heme-containing peroxidases, exists in neutrophils and monocytes. MPO has a broad spectrum of microbial killing. The difficulty of producing MPO at a large scale hinders its study and utilization. This study aimed to overexpress recombinant human MPO and characterize its microbicidal activities *in vitro* and *in vivo*. A human HEK293 cell line stably expressing recombinant MPO (rMPO) was established as a component of this study. rMPO was overexpressed and purified for studies on its biochemical and enzymatic properties, as well as its microbicidal activities. In this study, rMPO was secreted into culture medium as a monomer. rMPO revealed enzymatic activity similar to that of native MPO. rMPO, like native MPO, was capable of killing a broad spectrum of microorganisms, including Gram-negative and -positive bacteria and fungi, at low nM levels. Interestingly, rMPO could kill antibiotic-resistant bacteria, making it very useful for treatment of nosocomial infections and mixed infections. The administration of rMPO significantly reduced the morbidity and mortality of murine lung infections induced by Pseudomonas aeruginosa or methicillin-resistant Staphylococcus aureus. In animal safety tests, the administration of 100 nM rMPO via tail vein did not result in any sign of toxic effects. Taken together, the data suggest that rMPO purified from a stably expressing human cell line is a new class of antimicrobial agents with the ability to kill a broad spectrum of pathogens, including bacteria and fungi with or without drug resistance.

**IMPORTANCE** Over the past 2 decades, more than 20 new infectious diseases have emerged. Unfortunately, novel antimicrobial therapeutics are discovered at much lower rates. Infections caused by resistant microorganisms often fail to respond to conventional treatment, resulting in prolonged illness, greater risk of death, and high health care costs. Currently, this is best seen with the lack of a cure for coronavirus disease 2019 (COVID-19). To combat such untreatable microorganisms, there is an urgent need to discover new classes of antimicrobial agents. Myeloperoxidase (MPO) plays an important role in host defense. The difficulty of producing MPO on a large scale hinders its study and utilization. We have produced recombinant MPO at a large scale and have characterized its antimicrobial activities. Most importantly, recombinant MPO significantly reduced the morbidity and mortality of murine pneumonia induced by Pseudomonas aeruginosa or methicillin-resistant Staphylococcus aureus. Our data suggest that recombinant MPO from human cells is a new class of antimicrobials with a broad spectrum of activity.

## INTRODUCTION

Infectious diseases are a leading cause of death worldwide, particularly in the young and the elderly. The World Health Organization (WHO) reported that two infectious diseases (lower respiratory infections and diarrheal diseases) ranked in the top 10 worldwide causes of death (fourth and eighth, respectively) in 2019. Lower respiratory system infectious diseases were responsible for 2.74 million deaths worldwide (1), while diarrheal diseases were associated with an estimated 1.3 million deaths annually (2). During the past 2 decades, over 20 new infectious diseases have emerged, whereas novel antimicrobial therapeutics emerge at a much less rapid rate (3). Additionally, a variety of microorganisms with antimicrobial resistance are quickly emerging and spreading, and this situation persists because of inappropriate use of antimicrobial therapeutics (4). Specifically, among these is severe acute respiratory syndrome coronavirus 2 (SARS-CoV-2), the pathogen which causes coronavirus disease 2019 (COVID-19) (5). Antibiotic resistance is emerging not only in bacteria, such as S. aureus and E. coli, but also in fungi, viruses, and parasites (6). Infections caused by resistant microorganisms often do not respond to conventional treatment, resulting in prolonged illness and greater risk of death. A high percentage of nosocomial infections are caused by highly antibiotic-resistant bacteria, such as methicillin-resistant Staphylococcus aureus (MRSA) and drug-resistant Streptococcus pneumoniae. Multidrug-resistant P. aeruginosa causes health care-associated pneumonia and bloodstream infections, especially in immunocompromised patients. Therefore, there is an urgent need to discover new classes of antimicrobial agents in this era of new and reemerging infectious diseases with increasing antibiotic resistance.

The family of heme-containing animal peroxidases (hPxs) plays an important role in host defense (see reviews in references 7 and 8). Eight members with distinct cell/tissue distribution have been identified in humans. These members include myeloperoxidase (MPO), eosinophile peroxidase (EPO), lactoperoxidase (LPO), thyroid peroxidase (TPO), Duox1/2, PXDN, and PXDNL (7, 9, 10). hPxs, with the exception of PXDNL, which does not activate peroxidase activity, utilize H_2_O_2_ to catalyze the oxidation of halides to generate hypohalous acids. The latter are potent oxidants and able to oxidize proteins, lipids, and DNA of microorganisms (7). Oxidation of proteins, lipids, and DNA leads to killing of microorganisms, although the exact mechanism remains to be elucidated. MPO, the prototype of the hPx family, is well-known in the study of innate host defense. MPO is synthesized in bone marrow early myeloid precursors and localizes in the azurophilic granules of neutrophils and monocytes (11). When neutrophils ingest microbes, azurophilic granules fuse with the nascent phagosome. MPO is released into the microorganism-containing compartment, where the activated phagocyte NADPH oxidase, also known as Nox2, generates superoxide. Superoxide is converted into H_2_O_2_ by superoxide dismutase (SOD) or simultaneously (12). MPO then catalyzes the oxidation of chloride to generate hypochlorous acid (HOCl) in the presence of H_2_O_2_. HOCl thereby contributes to killing ingested microbes.

Native MPO in the azurophilic granules of neutrophils is dimmer; each monomer contains a light chain and a heavy chain with molecular weights of 13.5 and 59 kDa, respectively (13). The plasma MPO constitutively secreted from bone marrow myeloid precursors is single chain, approximately 89 kDa, and is known as proMPO. Past research reported that recombinant MPO (rMPO) expressed in CHO cells was revealed as single chain with the same amino acid sequence as proMPO (14). rMPO from CHO cells has enzyme activity similar to that of the native MPO (14). rMPO derived from human cells has been studied in its processing and maturation, but not in antimicrobial activities (15). Here, we report the expression and production of rMPO in a human embryonic kidney cell line (HEK293), a widely used genetic engineering cell line in the pharmaceutical industry. rMPO kills bacteria and fungi at very low concentrations (nanomolar levels). Interestingly, rMPO also kills drug-resistant bacteria, such as MRSA and P. aeruginosa. rMPO shows great efficacy in the treatment of murine experimental pneumonia. During *in vitro* cytotoxicity assays and animal safety tests, the use of rMPO at up to 100 nM does not reveal signs of cell injury and inflammatory response. Our data suggest that rMPO is a candidate for a new class of antimicrobial agents with a broad spectrum of pathogens.

## RESULTS

### Expression, purification, and biochemical properties of rMPO.

**FIG 1:**
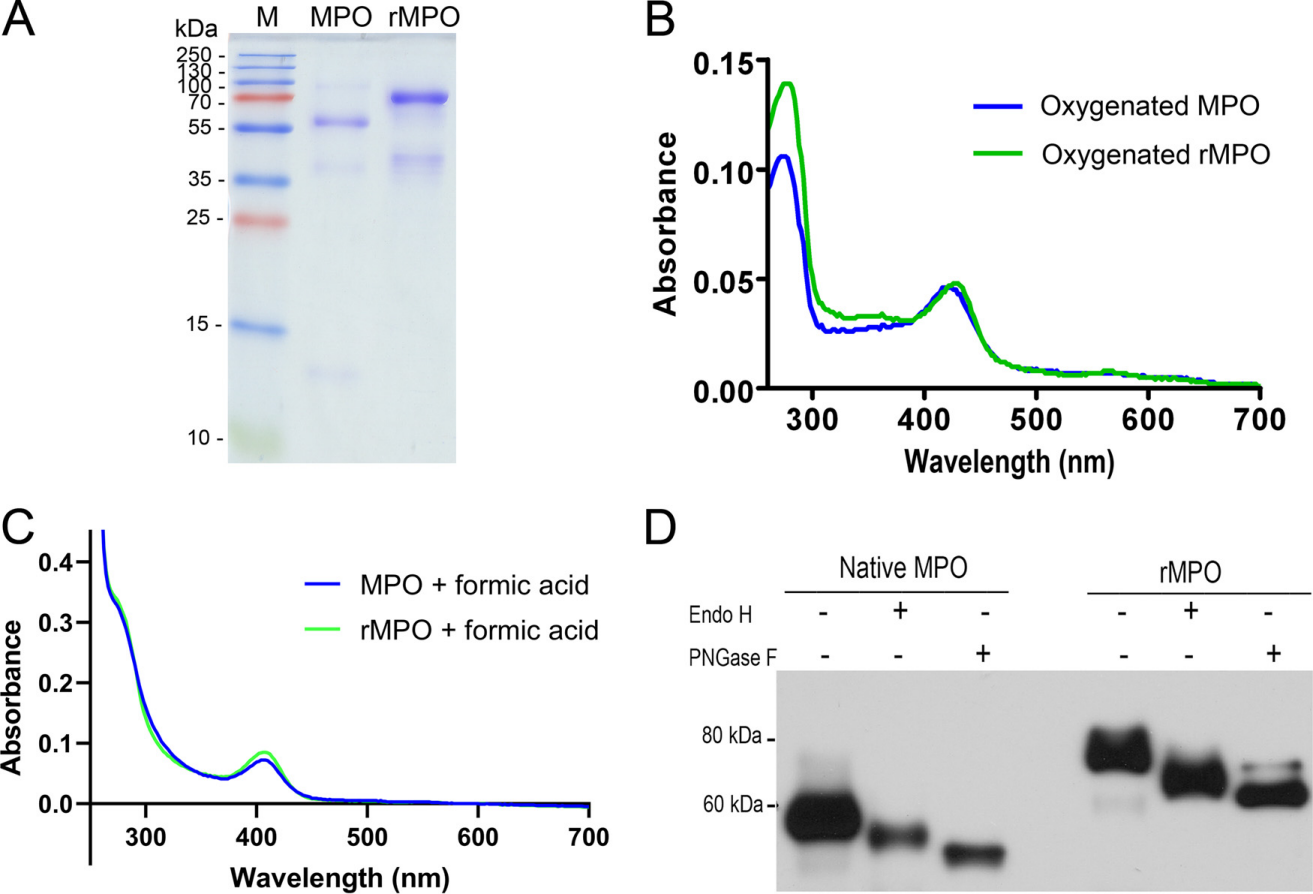
Purification of rMPO and its biochemical properties. (A) Purification of rMPO. rMPO was purified as described in Materials and Methods. A purified sample was run on 15% SDS-PAGE and stained by Coomassie blue. M, protein mass marker. The data shown are representative of more than three independent experiments. (B) UV-visible absorbance spectra of rMPO. rMPO was purified as described in the legend to panel A. The oxygenated spectra of rMPO and MPO (0.72 μM each) were recorded. The data shown are representative of three independent experiments. (C) Absorption spectra in 88% (vol/vol) formic acid were recorded. (C) Native MPO and rMPO were incubated in digestion buffer alone or digested with Endo H or PNGase F. Digests were separated by SDS-PAGE and transferred onto polyvinylidene difluoride (PVDF) membrane. Immunoblotting was carried out by using anti-MPO monoclonal antibody and visualized by chemiluminescence. The data shown are representative of two independent experiments.

The human MPO gene was stably overexpressed in HEK293 cells, and the protein secreted into the medium. The medium was subjected to two-step purification by using cation exchange resin and gel filtration. Unlike native MPO, which consists of heavy and light chains, rMPO showed a single band of 75 kDa (Fig. 1A). A weak band of approximately 42 kDa in the rMPO lane (Fig. 1A), which we assume is a result of rMPO degradation, was detected by anti-MPO monoclonal antibody (Fig. S2 in the supplemental material). The purity of the 75-kDa protein was 91%, as measured by ImageJ software. The ratio of absorbance at 430 nm to 280 nm (Reinheit Zahl [RZ] value, *A*_430_/*A*_280_) is 0.52. Two forms of rMPO protein (75 kDa and 90 kDa) were observed (Fig. S1). Fig. 1B shows the ferric-heme UV-visible absorbance spectra of the oxygenated native MPO and rMPO. rMPO has a sharp Soret absorbance peak maximum, at 430 nm (Fig. 1B). The spectrum of rMPO in formic acid is almost identical to the spectrum of MPO (Fig. 1C). These similar spectra indicate that native MPO and rMPO have overall similar absorbance spectra and heme loading. Endoglycosidase H (Endo H) treatment resulted in a molecular weight of rMPO from 75 to 68 kDa (reduction of 7 kDa), while MPO’s molecular weight changed from 59 kDa to 52 kDa (reduction of 7 kDa). Peptide-*N*-glycosidase F (PNGase F) treatment caused a molecular-weight change of rMPO from 75 kDa to 65 kDa (reduction of 10 kDa), while MPO changed from 59 kDa to 49 kDa (reduction of 10 kDa). Endo H cleaves within the chitobiose core of high-mannose and some hybrid oligosaccharides, while PNGase F cleaves between the innermost GlcNAc and asparagine residues of high-mannose, hybrid, and complex oligosaccharides from N-linked glycoproteins. Thus, Endo H and PNGase F cleave the same weights of carbohydrate contents in native MPO and rMPO (i.e., 7 kDa and 10 kDa, respectively) (Fig. 1D and Fig. S2). Taken together, these data suggest that rMPO is a 75-kDa monomer with biochemical properties similar to those of native MPO.

### Enzymatic properties of rMPO.

**FIG 2:**
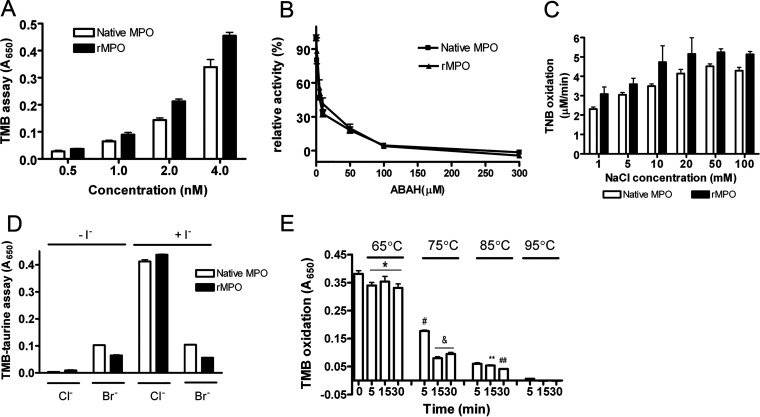
Enzymatic properties of rMPO. (A) TMB oxidation was carried out as described in Materials and Methods, in triplicate. The TMB liquid substrate system was used as the substrate. (B) TMB oxidation was carried out as described in the legend to panel A with the inhibitor of heme peroxidases, ABAH, in triplicate. (C) TNB oxidation was carried out as described in Materials and Methods, in triplicate. (D) Taurine-Cl generation coupling TMB oxidation by rMPO in triplicate. (E) Thermal stability of rMPO. rMPO was first incubated at the indicated temperatures for the indicated times. Then, the sample was used for TMB assay as described in the legend to panel A, in triplicate. Student’s unpaired *t* test; *P* < 0.05: *, 65°C versus control; #, 75°C for 5 min versus control; &, 75°C for 15 and 30 min versus 75°C for 5 min; **, 85°C for 15 min versus 85°C for 5 min; ##, 85°C for 30 min versus 85°C for 15 min. Data in all panels are representative of at least three independent experiments. Since different batches of native MPO have different activity units, these assays aim to determine if rMPO has peroxidase activities. Statistical analyses for the exact comparison of MPO and rMPO activities are not necessary. Error bars show standard deviations.

We carried out experiments to compare the enzymatic activities of rMPO and native MPO. rMPO showed activity similar to that of MPO in 3,3′,5,5′-tetramethylbenzidine (TMB) oxidation (Fig. 2A). The activity was inhibited by 4-aminobenzoic acid hydrazide (ABAH) in a dose-dependent manner (Fig. 2B). The oxidation of 5-thio-2-nitrobenzoic acid (TNB) by rMPO-mediated HOCl was consistently similar to that of native MPO (Fig. 2C). Like native MPO, rMPO catalyzed with a similar taurine oxidation (Fig. 2D). Table 1 summarizes the kinetic parameters of HOCl and hypobromous acid (HOBr) generation by native MPO and rMPO. We further determined the thermal stability of rMPO by incubation of rMPO at 65, 75, 85, or 95°C for 5, 15, or 30 min. rMPO only lost 13% of its activity when incubated at 65°C for 30 min (Fig. 2E). Increasing the temperature caused rapid loss of rMPO activity. Incubation of rMPO at 75°C for 5 min led to the loss of 50% of its activity, while incubation at 95°C for 5 min completely inactivated rMPO (Fig. 2E). These results are like those for native MPO (14).

**TABLE 1:** The enzymatic kinetics of HOCl and HOBr generation by native MPO and rMPO^*a*^

Form of MPO	Substrate	*K_m_* (μM)	*V*_max_ (μM/min)	*K*_cat_ (s^−1^)	*K_X_*^−^ (M^−1^ · s^−1^)
Native	NaCl	3,237.6 ± 128.1	5,445.3 ± 229.9	4,537.7 ± 191.6	1.4 × 10^6^ ± 7.4 × 10^4^
	KBr	3.50 ± 0.21	3.67 ± 0.18	3.06 ± 0.15	8.8 × 10^5^ ± 3.4 × 10^4^

Recombinant	NaCl	3,621.1 ± 446.1	6,242.2 ± 769.1	5,201.8 ± 640.9	1.4 × 10^6^ ± 1.7 × 10^5^
	KBr	3.31 ± 0.02	3.89 ± 0.03	3.24 ± 0.02	9.8 × 10^5^ ± 7.3 × 10^3^

a A TNB assay was carried out for measurement of kinetics of native MPO and rMPO as follows: 20 nM MPO was mixed with 50 mM potassium phosphate buffer, pH 5.4, containing 100 μM H_2_O_2_, 100 μM TNB, and NaCl (1, 5, 10, 20, 50, or 100 mM) or KBr (10, 20, 50,100, 200, or 500 μM). The Δ*A*_412_ was calculated. The maximum rate of reaction (*V*_max_) and the *K_m_* were determined according to the Lineweaver-Burk equation. The catalytic rate constants (*K*_cat_) were calculated by dividing the *V*_max_ value by the concentration of MPO. The specificity constants (*K_X_*^−^) were determined by dividing the *K*_cat_ by the *K_m_* value. The data are the mean results of two independent experiments.

### Bactericidal activities.

**FIG 3:**
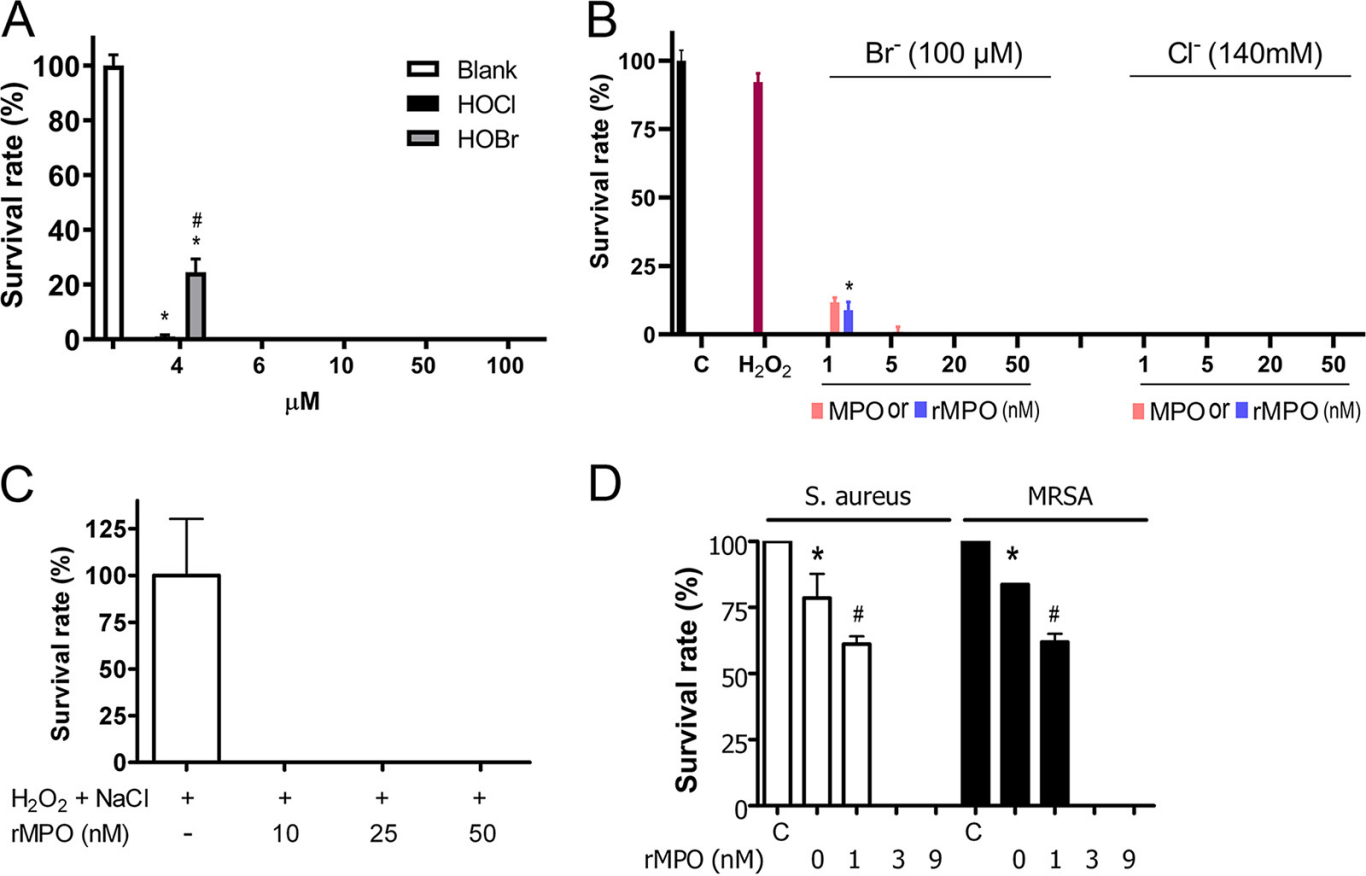
Bactericidal activities of rMPO. (A) Bactericidal activities of reagent hypohalous acids. E. coli was incubated in 50 mM potassium phosphate buffer, pH 7.4, and the indicated amount of reagent HOBr or HOCl at 37°C for 1 h. The mixture was plated on LB plates in triplicate. The plates were placed at 37°C overnight. CFU were counted. The survival rate (%) is expressed as the count for the experimental group divided by the count for the control group. Student’s unpaired *t* test analysis: *, *P* < 0.05, HOCl or HOBr versus control; #, *P* < 0.05, HOCl versus HOBr at 4 μM. (B) Dose-dependent MPO-mediated E. coli killing. E. coli was mixed into 50 mM potassium phosphate buffer, pH 6.2, 20 μM H_2_O_2_, and 140 mM NaCl or 100 μM KBr. MPO or rMPO was added as indicated. The mixture was incubated at 37°C for 1 h. The mixture was plated on LB plates in triplicate. CFU were counted after incubation at 37°C overnight. Survival rate (%) was calculated as the count for the experimental group divided by the count for the control group, which was E. coli only. Student’s unpaired *t* test analysis: *, *P* < 0.05, rMPO versus MPO at 1 nM. (C) P. aeruginosa was incubated in 50 mM potassium phosphate buffer, pH 7.4, containing 140 mM NaCl, 20 μM H_2_O_2_, and the indicated amount of rMPO at 37°C for 1 h. In the control experiment, only H_2_O_2_ and Cl^−^ were present. The survival rate (%) was calculated as described in the legend to panel A. Each experiment was performed in triplicate. (D) S. aureus or MRSA was incubated in 50 mM potassium phosphate buffer, pH 7.4, containing 140 mM NaCl, 20 μM H_2_O_2_ and the indicated amount of rMPO, at 37°C for 1 h. S. aureus or MRSA only was used as the control (C). The survival rate (%) was calculated as described in the legend to panel A. Each experiment was performed in triplicate. Student’s unpaired *t* test analysis: *, *P* < 0.05, 0 nM rMPO versus control; *#*, *P* < 0.05, 1 nM rMPO versus 0 nM. (A and B) Data are representative of two independent experiments. (C and D) Data are representative of three independent experiments. Error bars show standard deviations.

Four representative bacteria, including Gram-negative and -positive bacteria and drug-resistant bacteria, were used in the experiments. E. coli and P. aeruginosa are Gram-negative bacteria, while S. aureus and MRSA are Gram-positive bacteria. P. aeruginosa strain K is intrinsically multiple-drug resistant, while MRSA is methicillin resistant. First, we characterized the bactericidal activities of reagents HOBr and HOCl (Fig. 3A). A concentration of 4 μM reagent HOBr killed 72% of E. coli cells, while 4 μM reagent HOCl killed 99%. The bactericidal activities of MPO were dose dependent. A concentration of 1 nM MPO or rMPO in the MPO/H_2_O_2_/Cl^−^ (100 mM) system was enough to kill all E. coli cells, while 5 nM MPO or rMPO in the MPO/H_2_O_2_/Br^−^ (100 μM) system could kill all E. coli cells (Fig. 3B). These results are consistent with the oxidant potentials of the respective halides (HOCl > HOBr). Additionally, we carried out a further P. aeruginosa killing experiment using rMPO. As shown by the results in Fig. 3C, rMPO completely killed P. aeruginosa cells at 10 nM. We also carried out bactericidal activities for Gram-positive bacteria and drug-resistant bacteria. Similar to the results for Gram-negative bacteria, rMPO efficiently killed both S. aureus and MRSA at the nanomolar level (Fig. 3D). Thus, the data suggest that rMPO has powerful bactericidal activity.

### Fungicidal activities.

**FIG 4:**
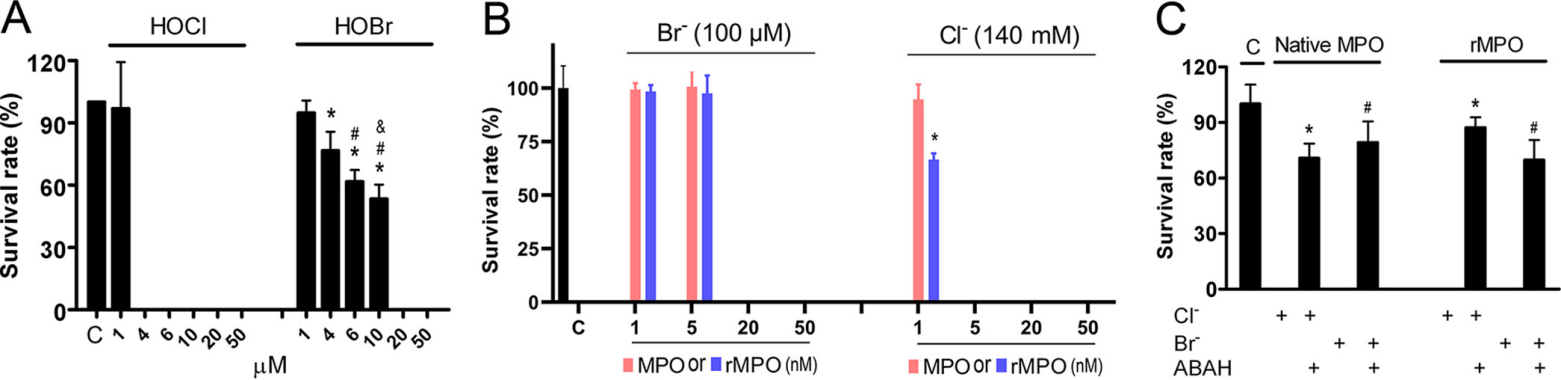
Fungicidal activities of rMPO. (A) Fungicidal activity of reagent HOCl and HOBr. C. albicans was incubated in 50 mM potassium phosphate buffer, pH 6.2, and the indicated amount of reagent HOBr or HOCl at 37°C for 1 h. The mixture was plated on YPD plates in triplicate. In control experiments, no hypohalous acid was present. The survival rate was calculated. Student’s unpaired *t* test analysis: *, *P* < 0.05, HOBr versus control; #, *P* < 0.05, HOBr at 6 μM versus HOBr at 4 μM; &, *P* < 0.05, HOBr at 10 μM versus HOBr at 6 μM. (B) Dose-dependent MPO-mediated C. albicans killing. C. albicans was incubated in 50 mM potassium phosphate buffer, pH 6.2, containing 100 mM NaCl or 100 μM KBr and 50 nM rMPO or MPO at 37°C for 1 h. The reactions were initiated by the addition of 50 μM H_2_O_2_. Cell mixture was plated on YPD plates in triplicate. CFU were counted after incubation at 37°C overnight. Control was C. albicans only. Survival rate (%) was calculated. Student’s unpaired *t* test analysis: *, *P* < 0.05, 1 nM rMPO versus 1 nM MPO for Cl^−^. (C) C. albicans killing was carried out as described in the legend to panel B. ABAH (300 μM) was added in some experiments. Cell mixtures were plated on YPD plates in triplicate. Survival rate (%) was calculated. Student’s unpaired *t* test analysis:, *, *P* < 0.05, MPO or rMPO with Cl^−^ versus MPO or rMPO with Cl^−^ plus ABAH; *#*, *P* < 0.05, MPO or rMPO with Br^−^ versus MPO or rMPO with Br^−^ plus ABAH. (A and B) Data are representative of two independent experiments. (C) Data are representative of three independent experiments. Error bars show standard deviations.

As shown by the results in Fig. 4A, 4 μM reagent HOCl or 20 μM reagent HOBr completely killed C. albicans cells. We then compared the C. albicans killing activity of rMPO with that of native MPO in the presence of H_2_O_2_ and the physiological concentration of Cl^−^ or Br^−^. In the dose-dependent experiments, using 140 mM Cl^−^ plus 50 μM H_2_O_2_ as the substrate, 5 nM rMPO or MPO completely killed C. albicans cells, whereas with 100 μM Br^−^ plus 50 μM H_2_O_2_ as the substrate, 5 nM rMPO or native MPO did not have fungicidal activity (Fig. 4B). A concentration of 20 nM rMPO or native MPO with 100 μM Br^−^ plus 50 μM H_2_O_2_ revealed complete fungal killing (Fig. 4B). The fungicidal activities of both rMPO and MPO were inhibited by ABAH (Fig. 4C). Thus, rMPO and MPO had similar fungicidal activities. Collectively, rMPO, like native MPO, could kill both bacteria and fungi. rMPO was functionally undistinguishable from native MPO.

### Cytotoxicities of rMPO and H_2_O_2_.

**FIG 5:**
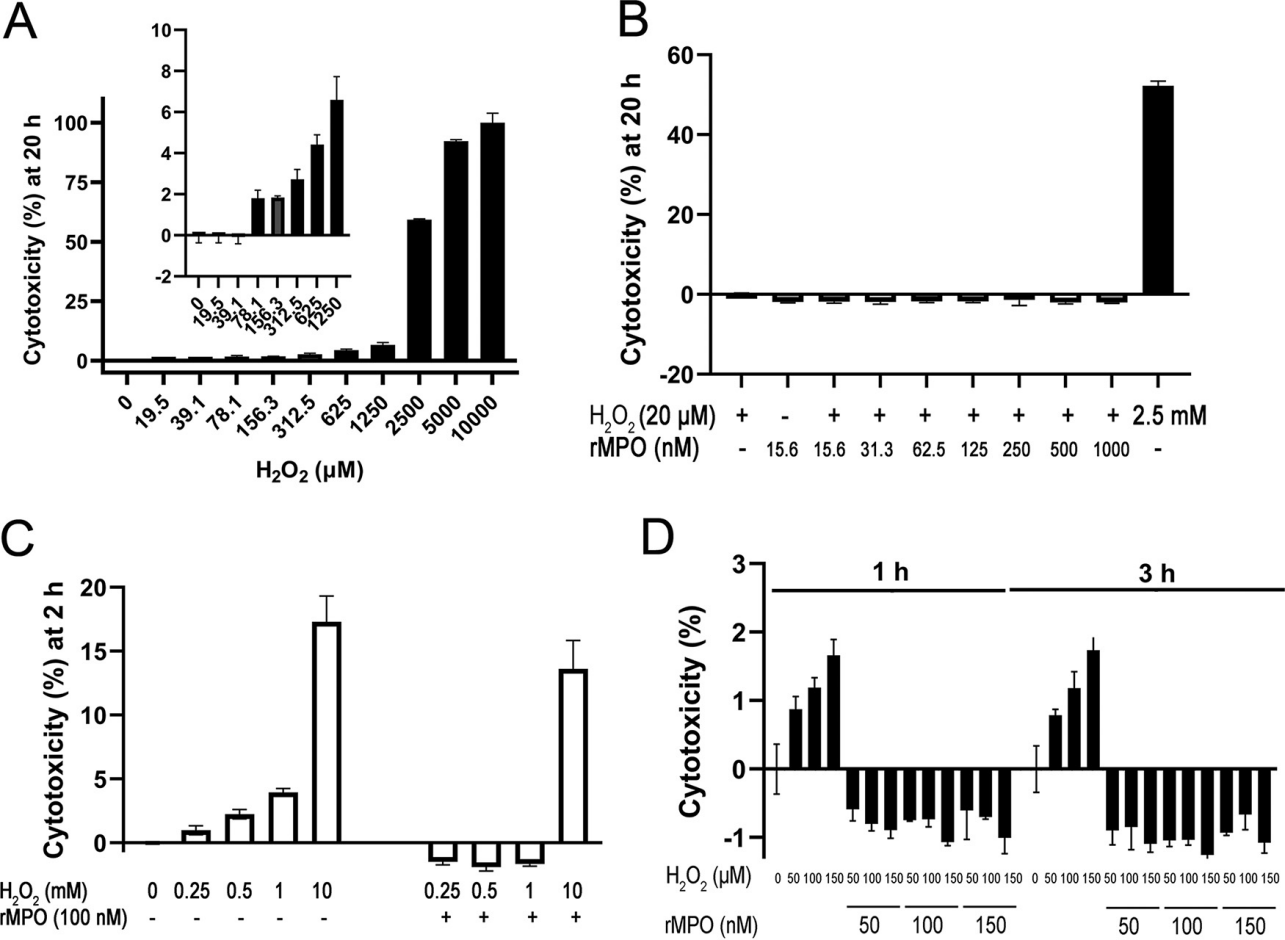
Cytotoxicity assay of H_2_O_2_ and rMPO. A549 cells grew in 100 μL of DMEM medium/10% FBS with 100 IU penicillin and 100 μg/mL streptomycin in a 96-well plate (2.5 × 10^4^/well). The next day, H_2_O_2_ and/or rMPO was added to indicated concentrations. After the indicated times, LDH activity was assayed using the CyQuant LDH cytotoxicity assay kit. Absorbance at 490 nm was measured. Percentage of cytotoxicity was calculated by the following equation: (*A*_490_ of treated LDH activity − *A*_490_ of LDH activity of PBS control)/(*A*_490_ of maximum LDH activity − *A*_490_ of LDH activity of PBS control) × 100. (A) Dose-dependent H_2_O_2_-mediated cytotoxicity at 20 h of incubation. Inset is lower concentrations of H_2_O_2_. EC_50_ is 2,280 μM. (B) rMPO plus 20 M H_2_O_2_. The cells were incubated for 20 h. PBS was the negative control, while 2.5 mM H_2_O_2_ was the positive control. (C) A concentration of 100 nM rMPO plus high concentrations of H_2_O_2_ as indicated were added into the cell mixtures. The cells were incubated for 2 h. ANOVA, *P* < 0.05. (D) rMPO and H_2_O_2_ were added into the cell mixtures as indicated. When the cells had been incubated for 1 and 3 h, part of the culture medium was taken for the assay. ANOVA, *P* > 0.05, rMPO versus controls; 1 h versus 3 h. (A to D) The data are representative of two independent experiments. Error bars show standard deviations.

As shown by the results in Fig. 5A, H_2_O_2_ possessed dose-dependent cytotoxicity for A549 cells. The concentration that resulted in 50% cytotoxicity (50% effective concentration [EC_50_]) was 2,280 μM. The concentration that resulted in 5% cytotoxicity (EC_5_) was 120 μM, while 2% cytotoxicity (EC_2_) was seen at 47 μM at 20 h of incubation. A concentration of 20 μM H_2_O_2_ plus rMPO up to 1 μM did not cause cytotoxicity (Fig. 5B). We further examined higher H_2_O_2_ concentrations with 100 nM rMPO, the latter being used for the animal study. As shown by the results in Fig. 5C, higher concentrations of H_2_O_2_ mediated cytotoxicity. Surprisingly, 100 nM rMPO significantly decreased H_2_O_2_-mediated cytotoxicity (Fig. 5C). A concentration of 100 nM rMPO could completely inhibit 1 mM H_2_O_2_-mediated cell injury while significantly reducing 10 mM H_2_O_2_-mediated cell injury (Fig. 5C). Further experiments showed that rMPO from 50 to 150 nM plus H_2_O_2_ from 50 to 150 μM did not cause cell damage (Fig. 5D). Taken together, H_2_O_2_ at 120 μM resulted in 5% cytotoxicity in A549 cells at 20 h. rMPO up to 1 μM did not mediate cytotoxicity. In contrast, rMPO could protect cells from H_2_O_2_-mediated cell injury.

### Animal safety of rMPO.

**FIG 6:**
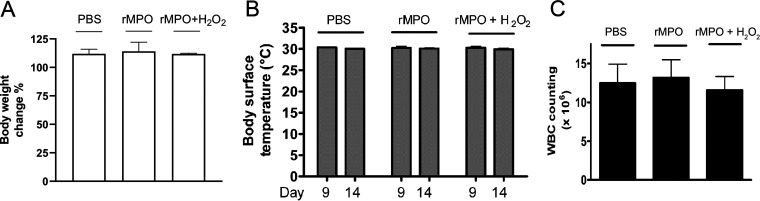
Animal safety test of rMPO. Three groups of C57BL/6 mice (6/group, male and female, 6 to 8 weeks old) were administered PBS, rMPO (100 nM) or rMPO (100 nM) plus H_2_O_2_ (20 μM), respectively, daily for 6 days via tail vein. The mice were observed for mortality, along with measurements for body weight, body surface temperature, and WBCs for up to 14 days. (A) Percentages of body weight change from day 0 to day 9. ANOVA, *P* > 0.05. (B) Body surface temperatures. ANOVA, *P* > 0.05. (C) WBC counts. ANOVA, *P* > 0.05. (A to C) The data are representative of two independent experiments. Error bars show standard deviations.

All mice survived after the administration of 100 nM (final plasma concentration) rMPO for 6 days. The mean body weight and body surface temperature of the mice did not undergo significant changes (Fig. 6A and B). The numbers of white blood cells (WBCs) at day 14 were similar in the control and treatment groups (Fig. 6C). The data indicated that intravenous (i.v.) administration of rMPO at 100 nM in blood for 6 days did not cause any signs of toxic effects.

### Treatment of acute lung infections by rMPO.

**FIG 7:**
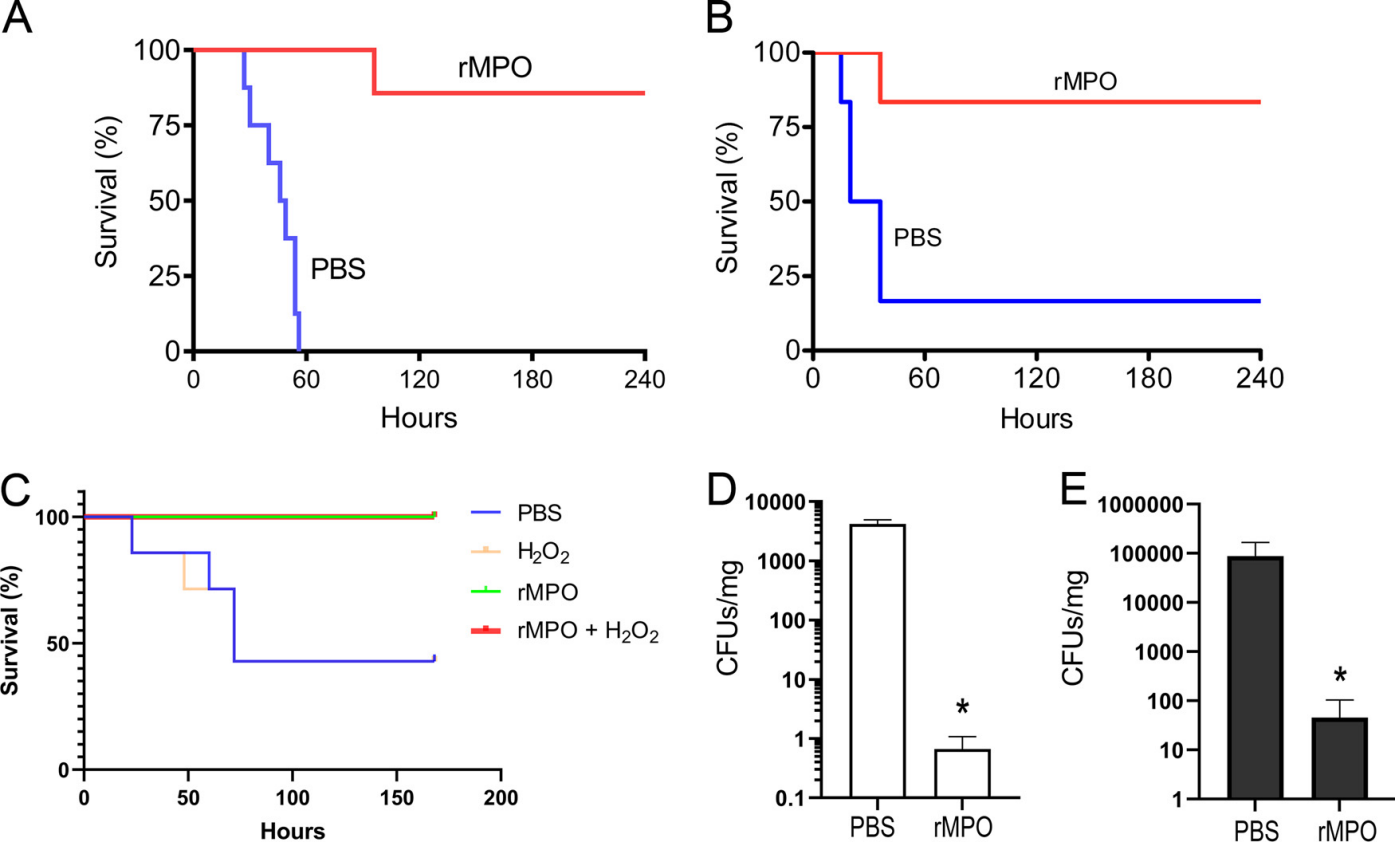
Efficacy of rMPO in treatment of acute lung infections. (A) The survival rate (%) of mice with P. aeruginosa infection. Acute lung infection of C57BL/6 mice by P. aeruginosa was carried out as described in Materials and Methods. Mice were treated by intratracheal instillation with 40 μL PBS containing 100 nM rMPO and 20 μM H_2_O_2_. Control group was treated with PBS only. *n* = 7 or 8. *P* = 0.0001. (B) The survival rate (%) of mice with MRSA infection. *n* = 6; *P* = 0.0178. (C) The survival rate (%) of mice with infection with P. aeruginosa and administration of rMPO 3 h apart. Acute lung infection of mice by P. aeruginosa was carried out as described in Materials and Methods. After 3 h, mice were intratracheally instilled with 50 μL PBS containing 100 μM H_2_O_2_, 200 nM rMPO, or 200 nM rMPO plus 100 μM H_2_O_2_. The control group was treated with 50 μL PBS. Log-rank test, *P* = 0.0130; Log-rank test for trend, *P* = 0.0035; *n* = 7. (D and E) Bacterial burdens of the lungs of mice in the experiments whose results are shown in panels A and B, respectively. After 24 h, lungs from control or rMPO-treated mice were weighed and homogenized. Tissue suspensions were diluted at 1:100 and 1:10,000 or not diluted, and 50 μL of suspensions were plated on LB agar in triplicate. CFU were counted, and bacterial burdens were expressed as CFU/mg tissue. *n* = 3 mice; *P* < 0.05. Error bars show standard deviations.

We further tested the efficacy of rMPO in the treatment of murine lung infections. P. aeruginosa- and MRSA-induced acute lung infections were utilized as disease models. A single administration of 100 nM rMPO in 40 μL had significant effects on animal survival. The survival rates after administration of rMPO for P. aeruginosa*-* and MRSA-infected mice were 85.7% and 83.3%, respectively. All untreated P. aeruginosa*-*infected mice died within 60 h, and in the same period, 83.3% of untreated MRSA-infected mice died (Fig. 7A and B). rMPO plus H_2_O_2_ treatment after 3 h postinfection significantly improved survival (Fig. 7C). Interestingly, rMPO alone had the same efficacy as rMPO plus H_2_O_2_, indicating the lung had enough H_2_O_2_ for rMPO during infection (Fig. 7C). The microbial burdens of the lungs infected with P. aeruginosa and MRSA at 24 h postinfection were examined. The lung suspensions from control groups of both bacterial infections had significantly more CFU per mg tissue than those of rMPO-treated groups (Fig. 7D and E). The average CFU count per mg tissue for P. aeruginosa infection was ∼4,000, while for MRSA, it was ∼100,000. The discrepancy was assumed to be due to the different CFU counts used in the infections. These data suggest that rMPO is excellent for treatment of P. aeruginosa- and MRSA-induced pneumonia. Taken together, the present study provides strong evidence that rMPO has great potential as a new class of antimicrobial agents.

## DISCUSSION

During the past 2 decades, more than 20 new infectious diseases have emerged. In addition, the growing antimicrobial resistance is a global problem. The old infectious diseases, which were previously thought to be controlled, have rebounded and regrouped. New and reemerging infectious agents, including drug-resistant forms, continue to pose serious threats. The latest report from the Centers for Disease Control and Prevention in the United States estimated that in 2017, methicillin-resistant S. aureus caused 323,700 hospitalizations and 10,600 deaths, resulting in $1.7 billion in health care costs. Furthermore, our existing antimicrobial agents rapidly lose their effectiveness due to multidrug-resistant pathogens, and there appear to be few, if any, new classes of drugs currently in clinical development. These trends increasingly challenge public health and medical care professionals.

Although most infectious diseases can be prevented and cured, numbers of infectious diseases still lack efficient approaches for treatment, particularly for drug-resistant pathogens. The discovery of new antibiotics has reached a plateau, while multidrug-resistant pathogens dismiss the effects of previously effective drugs. MPO plays an important role in host defense. MPO can kill a variety of pathogens (7, 16). There were attempts to produce rMPO from Sf9 insect cells (17), CHO cells (14, 18), and HEK293 cells (15). However, the low yields, distinct carbohydrate contents, and immunogenicity are concerning for the clinical use of rMPO. Herein, we report the overexpression and purification of rMPO derived from human cells and the characterization of its biochemical and enzymatic properties, as well as its microbicidal activities. rMPO is a monomer with a carbohydrate content similar to those of native monomers. Unlike most proteins, it has excellent thermal stability. Remarkably, at 100 nM, rMPO can kill a broad spectrum of microorganisms, including bacteria, drug-resistant bacteria, and fungi, without showing cell injury.

The conventional antibiotics are chemical compounds, most of which inhibit either critical proteins in specific metabolisms or microorganism transportation. In addition, bacteria readily develop resistance to antibiotics. MPO is an enzyme with antimicrobial activity that plays an important role in innate immunity. It selectively kills pathogens via the generation of HOCl (19). HOCl oxidizes important components of pathogens, including proteins, DNA, and lipids. There is no report that microbes, including P. aeruginosa and MRSA, have developed resistance to MPO or HOCl.

Research estimates that healthy human adults synthesize approximately 2.8 μmol (i.e., 0.4 g) of MPO per day (19). Isolating MPO from blood is either technically difficult or uneconomic at a large scale. This hinders the use of MPO as an antimicrobial agent in treating infectious diseases. The production of recombinant proteins in human cells has many advantages, such as large yields, suitable posttranscriptional modification, integration of the appropriate cofactors, and protein secretion. Human HEK293 cells have been extensively used for the large-scale production of recombinant eukaryotic proteins (20). We have established a cell line derived from HEK293 cells that stably expresses MPO and developed a series of techniques for overexpression and purification of rMPO, as well as characterizing its enzymatic properties and microbicidal activities. Thus, rMPO is an excellent candidate for a new class of antimicrobial agents, providing a great opportunity for treatment of a broad spectrum of infectious diseases, including bacterial, viral, and fungal infections.

Glycosylation machinery often adds undesired carbohydrate determinants, which may alter protein folding, induce immunogenicity, or reduce the circulatory life span of recombinant enzymes or protein drugs. Glycosylation is cell-type specific, and different host cells contain different patterns of oligosaccharides that may affect biological functions (21). Notably, sialic acid as *N*-acetylneuraminic acid is not efficiently added in most mammalian cells, and the 6-linkage is missing in rodent cells (21). The carbohydrate contents of rMPO from CHO cells are largely unknown. CHO cells cannot provide a human-like pattern of glycosylation to recombinant proteins (22). Thus, it is assumed that rMPOs from CHO cells and HEK293 cells have distinct carbohydrate contents. This discrepancy could cause differences in enzymatic activities and immunogenicities. In terms of clinical use, rMPO from human cells, with less possibility of antigenicity, is much better than that from CHO cells.

The 90-kDa form of rMPO from HEK293 cells has been reported previously (15). Our study detected two forms (75 and 90 kDa) in the medium of cells stably expressing MPO. The 90-kDa protein is proMPO, while the 75-kDa protein is an intermediate (23). In neutrophils, most 90-kDa proteins (proMPO) undergo proteolytic processing by proprotein convertases to form the 75-kDa protein. The 75-kDa protein subsequently undergoes the endoproteolytic removal of a hexamer (ASFVTG) to produce the subunits of mature MPO in azurophilic granules. A small portion of proMPO proteins is secreted into the extracellular space (23). The fate of the proMPO proteins in the extracellular space is largely unknown. It is reported that the 90-kDa rMPO is the predominant form prepared in HEK293 cells (15). However, the present studies show that rMPO expressed in HEK293 cells is secreted into the medium and most proMPO proteins from the culture medium undergo proteolytic processing to form the 75-kDa protein as a predominant form. The previously reported dominant 90-kDa form (15) was prepared from the medium of a culture with ∼80% cell confluence, while the dominant 75-kDa form in the present study is from a culture with 100% cell confluence. It is reported that proprotein convertases (PCSK5, -6, and -9) are attached at the cell surface, while the soluble PCSK1, -2, -3, and -8 are released into the extracellular matrix (24). The Human Protein Atlas (www.proteinatlas.org) shows that HEK293 cells express PCSK3, -5, -6, and -8 (expression of PCSK6 > PCSK3 > PCSK8 > PCSK5). Therefore, we predict that these proprotein convertases are involved in the proteolytic processing of the 90-kDa to the 75-kDa rMPO in the culture medium of HEK293 cells.

The RZ value is a ratio of the heme Soret absorbance to the absorbance at 280 nm (*A*_430_/*A*_280_). Several research articles use the RZ value as the purity index for the MPO product. An RZ value of >0.8 is considered to show highly pure MPO (25, 26). rMPO prepared from CHO cells to a purity of 99% had an RZ value of ∼0.6 (26). The RZ value of rMPO in Fig. 1A was 0.52, and the purity was 91%. Therefore, the RZ value as a purity index is different for native MPO and rMPO. This could be caused by the different molecular structures (26).

rMPO catalyzes the same reactions as native MPO, including oxidation of Cl^−^ to generate HOCl. rMPO is very active, meaning a low concentration (5 nM) is enough to kill all bacteria and fungi. Fungi are eukaryotic and have both biochemical pathways and cellular machinery that are similar to those of their host. It is relatively difficult to find a drug that kills fungi without side effects. In addition, the thicker cell walls of fungi protect them from chemical attacks or drug transportation. There are very few antifungal agents in clinical treatment for fungus infections. Our data demonstrate that rMPO has strong fungicidal activities and suggest it as a potential therapeutic for fungus infections.

The molecular mechanism of MPO-mediated microbicidal activities remains to be elucidated. It is widely accepted that the MPO-generated potent oxidant HOCl mediates the oxidation of a variety of microbial components. HOCl interferes with or damages microbial metabolism, replication, and reproduction. Allen and Stephens proposed that MPO may selectively bind to pathogens and generate OCl^−^ and ^1^O_2_^·^ in the presence of H_2_O_2_ (19). Combustive destruction of microbial components occurs by concentration of MPO on microbes (19). Studies on the minimum inhibitory concentration (MIC) and minimum bactericidal concentration (MBC) of MPO indicate that high MPO binding to pathogens is related to low MIC and MBC values of MPO. Thus, selective MPO binding results in selective MPO killing (19). MPO-generated HOCl, while possessing a powerful oxidation ability compared to that of chemical HOCl, is different from chemical HOCl by its production and release process (27).

Mammals have developed a series of approaches to prevent the negative consequences of oxidants or oxidant-generating enzymes while killing invading microbes. For instance, Chandler et al. reported that SCN^−^ is a dually protective molecule, able to both enhance host defense and decrease tissue injury and inflammation in lung infection (28). The inflammatory response and the concentration of MPO in bronchi of cystic fibrosis patients with infections were disproportionate (29). 1.4 -3.1 μM MPO were generally detected in sputum of these patients (29). Importantly, mammals possess sophisticated reduction and oxidation systems that can tightly regulate the reduction-oxidation reactions (30). It is reported that the median MPO serum level in the healthy elderly population is 258 pM and the top quartile is over 432 pM (31). In a study using MPO to predict the risk for acute coronary syndromes, MPO serum levels are up to ∼600 μg/liter (∼8 nM) (32). There are no data indicating how long the high levels of MPO exist. One may guess that the status may have already existed for months or years in these populations. Thus, when talking about negative consequences of MPO, one should consider the MPO concentration, time period, tissue location, H_2_O_2_ levels, the status of the antioxidant system, etc. In the present study, the data confirmed H_2_O_2_-mediated cytotoxicity in A549 cells. The EC_50_ was 2,280 μM in the conventional cell culture. The EC_5_ and EC_2_ of H_2_O_2_ were 120 μM and 47 μM, respectively, in 20 h of incubation. Therefore, less than 120 μM H_2_O_2_ for an *in vitro* cell assay for a cell signaling study should be beneficial, while more that amount will cause significant cell injury. Contrary to popular belief, our data indicated that rMPO protected cells from H_2_O_2_-mediated cell injury. In animal studies, i.v. administration of 100 nM rMPO for 6 days did not cause visible signs of negative consequences. Therefore, rMPO is safe for short-term administration at 100 nM.

In summary, we have established a system for the overexpression and purification of rMPO. rMPO has enzymatic properties and microbicidal activities similar to those of native MPO. Importantly, we demonstrate that rMPO is an effective agent for the treatment of experimental acute lung infections by P. aeruginosa and MRSA. Compared with conventional antibiotics, rMPO is an excellent candidate as a new antimicrobial agent not only because of its sensitivity but also its broad spectrum of antimicrobial activities.

## MATERIALS AND METHODS

### Microorganisms.

E. coli TOP10 was purchased from Life Technologies, Inc. (Grand Island, NY). Candida albicans strains were isolated from the hospital at the University of Alabama at Birmingham. P. aeruginosa strain K was a gift from Jean-Francis Pittet at the Department of Anesthesiology, University of Alabama at Birmingham (UAB). S. aureus (strain ATCC 25923, non-drug resistant) and methicillin-resistant S. aureus (strain ATCC BAA-1690) were purchased from American Type Culture Collection (ATCC, Manassas, VA).

### Reagents.

Luminol, bovine LPO, 3,3′,5,5′-tetramethylbezidine (TMB) and TMB liquid system, tyrosine, KBr, NaCl, 5-thio-2-nitrobenzoic acid (TNB), and 4-aminobenzoic acid hydrazide (ABAH) were purchased from Sigma-Aldrich (St. Louis, MO). Native human MPO was from Elastin Products Company, Inc. (Owensville, MO). pcDNA3.1, the CyQuant lactate dehydrogenase (LDH) cytotoxicity assay kit (catalog number C20300), and prestained protein markers were from Invitrogen (Carlsbad, CA). CM-Sepharose fast flow and Sephacryl S-300 were purchased from Cytiva (Marlborough, MA). Endoglycosidase H (Endo H) and peptide-*N*-glycosidase F (PNGase F) were from New England Biolabs (Ipswich, MA). Centrifugal filter devices (30-kDa cutoff) were from Millipore Corporation (Billerica, MA, USA). Anti-MPO monoclonal antibody (2C7) was purchased from Novus Biologicals (Centennial, CO). Pierce ECL Western blotting substrate was from ThermoFisher Scientific (Grand Island, NY).

### Establishing a cell line with expression of MPO.

Human full-length MPO was subcloned into pcDNA3.1(−), and the MPO sequence was verified by sequencing. The plasmid was transfected into HEK293 cells using the same procedures as described in reference 10. After 3 weeks of incubation with G418 (500 μg/mL), 10 G418-resistant colonies were isolated and grown in 10-cm plates. Peroxidase activities were measured by TMB oxidation assay, while MPO expression levels were determined by immunoblotting using anti-MPO antibody. The colony that expressed the highest level of MPO was selected and used for the production of rMPO.

### Production and purification of rMPO.

Stable MPO-expressing cells grew in 15-cm plates with Dulbecco’s Modified Eagle Medium (DMEM) containing 10% fetal bovine serum (FBS), 100 IU/mL penicillin, and 100 μg/mL streptomycin at 37°C under a 5% CO_2_ atmosphere. When the cells reached confluence, the medium was collected and centrifuged at 1,509 × *g* for 20 min. Two liters of the supernatant was loaded onto the column with 20 ml of CM-Sepharose Fast Flow and gradually eluted by 20 mM potassium phosphate buffer, pH 7.4, containing 0.1 M to 0.5 M NaCl. The eluent was collected in amounts of 3 mL/fraction. rMPO activity was monitored by TMB oxidation assay. The fraction with the higher activity was used for further purification. The eluent was then loaded onto a 2.5- by 80-cm Sephacryl S-300 column and eluted by 20 mM potassium phosphate at a rate of 0.5 mL/min. The eluent was collected in amounts of 4 mL/fraction. The protein concentration in the eluent fraction and the peroxidase activity were monitored by absorbance at 280 nm and 430 nm, respectively, as well as TMB oxidation assay. The eluent (8 mL) with the strongest peroxidase activity was collected.

### UV-Vis spectra.

The UV-Vis spectra of native MPO and rMPO were recorded from 260 to 700 nm using a UV2450 spectrophotometer (Shimadzu) according to the method described in reference 2. Absorption spectra were also recorded in 88% (vol/vol) formic acid, in which formic acid breaks the protein-heme interaction (33), facilitating comparison of the heme contents in MPO and rMPO.

### Measurement of heme concentration of rMPO.

The rMPO concentrations were expressed as the heme concentrations and calculated with the molar extinction coefficient at 430 nm of 89,000 M^−1^ cm.

### TMB oxidation assay for MPO activity.

The TMB liquid substrate system (catalog number T8665; Sigma-Aldrich) was used as the substrate to measure peroxidase activity as previously described (10). The absorbance at 650 nm was recorded. In some experiments, the peroxidase inhibitor ABAH was added.

### Taurine chlorination-TMB oxidation assay for HOCl generation.

HOCl generation was detected by utilizing the taurine chlorination assay in combination with TMB oxidation in the presence of iodide as previously reported (34), with slight modifications. In brief, the reaction was initiated by adding 50 μM H_2_O_2_ to 50 μl of 20 mM potassium phosphate buffer, pH 7.4, 140 mM NaCl (or 100 μM KBr), 5 mM taurine, and 20 nM rMPO or native MPO. The reaction mixture was incubated at 37°C for 30 min. The reaction was stopped by adding catalase (25 μg/mL). The reaction mixture was then mixed with freshly made developing agent. The developing agent consisted of 400 mM acetate buffer, pH 5.4, 1 mM TMB (predissolved in 100% dimethylformamide), and 100 μM NaI. After 5 min, absorbance at 650 nm was recorded. In some experiments, peroxidase inhibitors (ABAH and NaN_3_), H_2_O_2_ scavenger (catalase), and HOCl scavenger (methionine) were added as indicated. To selectively detect taurine chloramine, iodide was omitted in some reactions (34).

### Kinetics of MPO-mediated HOCl generation.

The enzymatic kinetics of rMPO- and native MPO-mediated HOCl generation were analyzed by using a TNB oxidation assay modified from previously reported studies (35). A concentration of 100 μM H_2_O_2_ was added into a mixture containing 20 mM potassium phosphate buffer, pH 5.5, 100 μM TNB, 20 nM native MPO or rMPO. NaCl was added to this mixture to initiate the reaction. The absorbance at 412 nm was immediately recorded every 30 s for 5 min. *V*_max_ and *K_m_* were obtained using a nonlinear least-square fit to the Michaelis-Menten equation.

### Analysis of glycosylation of rMPO.

One microgram of rMPO or native MPO was digested by Endo H or PNGase F following the manufacturer’s instructions at 37°C for 1 h. One-fourth of the reaction mixture was subjected to SDS-PAGE and immunoblotting analysis using anti-MPO antibody.

### Protein concentration.

Protein concentration was determined using Bio-Rad protein assay reagent based on the Bradford dye-binding procedure. Bovine serum albumin served as the protein standard.

### SDS-PAGE.

SDS-PAGE was carried out according to the standard method. Samples were mixed with SDS-PAGE sample loading buffer (50 mM Tris-HCl [pH 6.7], 2% [wt/vol] SDS, 20% [vol/vol] dithiothreitol [DTT], 10% [wt/vol] glycerol, and 0.05% bromophenol blue) and boiled for 3 min. Proteins were separated by electrophoresis in 15% (wt/vol) SDS-PAGE. Gels were subjected to staining with Coomassie brilliant blue or to immunoblot analysis.

### Immunoblot analysis.

Conventional immunoblot analysis was performed using anti-MPO monoclonal antibody. The second antibody was a horseradish peroxidase (HRP)-conjugated anti-mouse antibody. MPO was visualized by chemiluminescence using Pierce ECL Western blotting substrate.

### Bactericidal activity.

E. coli TOP10, P. aeruginosa K, and S. aureus strains (nonresistant strain ATCC 25923 and methicillin-resistant strain ATCC BBA-1690) were grown at 37°C overnight in LB broth or tryptic soy broth with shaking. Bacteria were washed three times with phosphate-buffered saline (PBS) before further experiments. A 100-μl mixture containing 50 mM potassium phosphate buffer, pH 7.4, 10^3^ to 10^4^ CFU bacteria, 140 mM NaCl, 20 μM H_2_O_2_, and the indicated amount of rMPO or native MPO was incubated at 37°C for 1 h. The cell mixture was plated on LB plates or tryptic soy agar plates in triplicate and incubated at 37°C overnight. CFU were counted. In control experiments, only H_2_O_2_ (20 μM) and/or Cl^−^ (140 mM) was present. The survival rate was expressed as the CFU count for the experimental group divided by the CFU count for the control group. In some experiments, ABAH was added to inhibit MPO.

### Fungicidal activity.

A single colony of C. albicans (UAB hospital isolate) grew in yeast extract-peptone-dextrose (YPD) broth at 30°C overnight with shaking. C. albicans was washed three times with PBS and then incubated in 50 mM potassium phosphate buffer, pH 6.2, containing 100 mM NaCl, 50 μM H_2_O_2_, and the indicated amount of rMPO or native MPO at 30°C for 1 h. Cell mixtures were plated on YPD plates in triplicate and incubated at 30°C overnight. In control experiments, only H_2_O_2_ (50 μM) or Cl^−^ (100 mM) was present. In some experiments, ABAH was added to inhibit MPO.

To verify either HOCl- or HOBr-mediated microbial killing, reagent HOCl or HOBr was utilized instead of MPO-generated HOCl or HOBr. HOCl was prepared and its concentration was determined as previously described (34), while HOBr was prepared by mixing NaOCl with KBr, as previously described (36). E. coli or C. albicans was incubated with 50 mM potassium phosphate buffer, pH 6.2, containing the indicated amount of HOCl or HOBr at 37°C (E. coli) or 30°C (C. albicans) for 1 h. Cell mixtures were plated on LB plates (E. coli) or YPD plates (C. albicans) in triplicate and incubated at 37°C (E. coli) or at 30°C (C. albicans) overnight. CFU were counted.

### Cytotoxicity assays of H_2_O_2_ and rMPO.

The lactate dehydrogenase assay detects LDH released from cells as a measure of cell membrane damage and cytotoxicity. A549 cells (adenocarcinomic human lung epithelial cell line) grew in 100 μL of DMEM 10% FBS with 100 IU/mL penicillin and 100 μg/mL streptomycin in a 96-well plate (2.5 × 10^4^/well). The next day, H_2_O_2_ and/or rMPO was added to the indicated concentrations. After the indicated times, LDH activity was assayed by using the CyQuant LDH cytotoxicity assay kit, following the manufacturer’s instructions. Absorbance at 490 nm was measured. The percentage of cytotoxicity was calculated by the following equation: (*A*_490_ of treated LDH activity − *A*_490_ of LDH activity of PBS control)/(*A*_490_ of maximum LDH activity − *A*_490_ of LDH activity of PBS control) × 100.

### Animal safety test of rMPO.

Three groups of C57BL/6 mice (6/group, male and female, 6 to 8 weeks old) were administered PBS, rMPO, or rMPO plus H_2_O_2_ (20 μM), respectively, daily for 6 days via the tail vein. rMPO was administered to 100 nM based on the approximate blood volume of a mouse with 77 to 80 μl blood/g (37). The mice were observed for mortality, body weight and body surface temperature were measured, and white blood cells (WBCs) were counted for up to 14 days. The body surface temperature of the back was measured by an infrared noncontact thermometer. Blood from mouse hearts was drawn at the 14th day to analyze the number of WBCs using an automated cell counter (ThermoFisher Scientific).

### Treatment of acute lung infections by rMPO.

The protocol was approved by the Institutional Animal Care and Use Committee of the University of Alabama at Birmingham. C57BL/6 mice (6 to 8/group) were anesthetized using 100 mg/kg of body weight ketamine–10 mg/kg xylazine intraperitoneally (i.p.). An amount of 7.5 × 10^6^ P. aeruginosa or 1.5 × 10^8^ MRSA in 40 μL PBS was instilled into each mouse’s trachea and immediately followed by 40 μL of PBS or 100 nM rMPO plus 20 μM H_2_O_2_. Animal survival was monitored for 10 days. In some experiments, P. aeruginosa and rMPO were administered 3 h apart. In brief, mice were anesthetized using 5% isoflurane, and 9.25 × 10^6^ P. aeruginosa in 40 μL PBS was instilled into the tracheas. After 3 h, mice were reanesthetized using 5% isoflurane, and 40 μL of PBS, 100 μM H_2_O_2_, 200 nM rMPO or 200 nM rMPO plus 100 μM H_2_O_2_ was instilled into the tracheas. Animal survival was monitored for 7 days. Notably, ketamine-xylazine i.p. would not have been appropriate to the latter experiment, in which mice twice encountered anesthesia.

### Burdens of P. aeruginosa and MRSA in the lung.

Mice were euthanized by CO_2_ 24 h postinfection. The bacterial clearance was carried out as described in reference 38. In brief, the mouse lung was aseptically removed and weighed. The lung tissue was then homogenized in cold sterile PBS. The tissue suspension was passed through a 100-μm sterile cell strainer. The suspension was centrifuged at 4,200 × *g* for 5 min. The pellet was washed twice with 1 mL PBS each time. After the last wash, the pellet was resuspended in PBS by adding 100 μL PBS per 10 mg tissue. The bacterial suspension was diluted at 1:100 and 1:10,000 with PBS. Amounts of 50 μL of diluted or nondiluted suspension were plated on LB agar (for P. aeruginosa) or tryptic soy broth agar (for MRSA) in triplicate. The plates were incubated at 37°C overnight, followed by a colony count.

### Statistics.

Data are shown as mean values ± standard deviations unless otherwise indicated. Quantitative variables were compared by means of Student’s unpaired *t* test for two groups or analysis of variance (ANOVA) for multiple groups. A *P* value of <0.05 was considered significant.
