# Supplementary material for: Recombinant Myeloperoxidase as a New Class of Antimicrobial Agents
Source: Microbiol Spectr. 2022 Jan 12;10(1):e00522-21. doi: 10.1128/spectrum.00522-21 (PMC8754140; doi:10.1128/spectrum.00522-21)
Supplement: SUPPLEMENTAL FILE 1 — Supplemental material. Download SPECTRUM00522-21_Supp_1_seq5.pdf, PDF file, 0.3 MB [file spectrum00522-21_supp_1_seq5.pdf]

Supplemental Materials for

Recombinant MPO as a new class of antimicrobial agents

Zehong Cao and Guangjie Cheng*

*Corresponding author: Email: guangjiecheng@uabmc.edu

SUPPLEMENTAL MATERIALS AND METHODS:

Control experiment for bactericidal activity

E. coli strain Top10 grew in LB broth at 37°C overnight with shaking. Bacteria were spun down at 4200 x g for 5 min. Bacteria were washed three times with PBS before further experiments. Bacteria (10^4 -10^5 CFU/ml) were incubated in 50 mM potassium phosphate buffer, pH 7.4, with or without containing 140 mM NaCl, 20 µM H_2O_2 and rMPO (50 nM) at 37 ºC for 1 h. Cell mixture was plated on LB plates in triplicates or quadruplicates. The plates were placed at 37ºC for overnight. CFUs were counted.

SUPPLEMENTAL FIGURES:

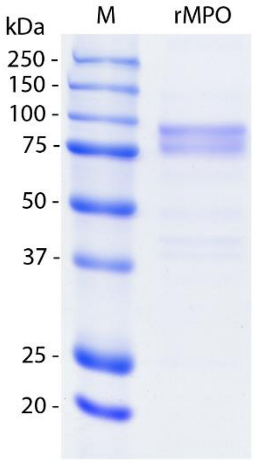

Supplemental Figure 1. Purified rMPO revealed two forms in some lots of purification. rMPO was purified as described in Materials and Methods. Purified sample was run on 15% SDS-PAGE and stained by Coomassie blue. M. Protein mass marker.

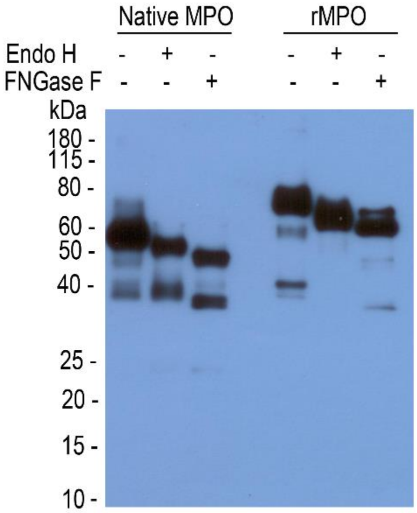

Supplemental Figure 2. Analysis of glycosylation of rMPO. The assay was as described in Materials and Methods. Native MPO and rMPO were incubated in digestion buffer alone or digested with Endo H or PNGase F. Digests were separated by SDS-PAGE and transferred onto PVDF membrane. Immunoblot analysis was carried out by using the anti-MPO monoclonal antibody (2C7). Some degraded bands are found. Native MPO shows a major degraded band at ~38 kDa and some weak bands including ~ 47 kDa. rMPO shows a major degraded band at ~42 kDa and two weak band at ~60 and 38 kDa respectively).

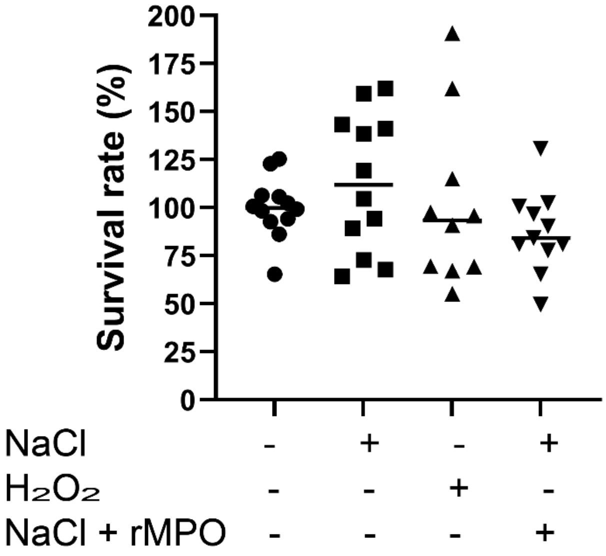

Supplemental Figure 3. Some control groups for bactericidal activity. *E. coli* was incubated in 50 mM potassium phosphate buffer, pH 7.4, with NaCl (140mM), H_2O_2 (50 µM) or rMPO (50 nM) as indicated at 37ºC for 1 h. The mixture was plated on LB plates in triplicate or quadruplicates. The plates were placed at 37ºC for overnight. CFUs were counted. The survival rate is calculated by divided the numbers of *E. coli* only group. The data are the combination of three independent experiments. One way of ANOVA, $p > 0.05$.
